# Supplementary material for: WordCommentsAnalyzer: A windows software tool for qualitative research
Source: F1000Res. 2018 Sep 4;7:536. Originally published 2018 May 3. [Version 2] doi: 10.12688/f1000research.14819.2 (PMC6137411; doi:10.12688/f1000research.14819.2)
Supplement: Supplementary file 3 [file f1000research-7-17614-s0002.tgz › b2a79496-4ada-47c4-9682-a99ce867836d.zip!/Eight Iranian Health Journals/The Journal of Toloo-e-Behdasht_17.docx]

© The Journal of Toloo-e-Behdasht. This work is licensed under CC BY 4.0 (https://creativecommons.org/licenses/by/4.0/).

Volume & Issue: دوره 14 - شماره 4

Title: تعیین مقادیر آلودگی فلزات سنگین در نمک‌های خوراکی موجود در بازار شهر اصفهان در سال 1389

http://tbj.ssu.ac.ir/article-1-1771-fa.html

مقدمه: عمده فلزات سنگین محیط شامل کادمیوم، آرسنیک، سرب و جیوه میباشند که مسئول بسیاری از ناهنجاری‌ها در انسان هستند و می‌توانند از طرق مختلف به زنجیره غذایی وارد شده، سلامت انسان را به مخاطره اندازند. در کنار آنها فلزاتی همچون روی و مس نیز وجود دارند که در غلظت‌های بالا اثرات مشابهی دارند. نمک طعام به عنوان اصلی ترین چاشنی در مواد غذایی می‌تواند حاوی این مواد غیر آلی باشد. هدف از این مطالعه تعیین آلودگی فلزات سنگین در نمک‌های مصرفی شهر اصفهان در سال1389بود. روش بررسی: در این مطالعه که از نوع توصیفی-مقطعی می‌باشد، تعداد 15نمک تصفیه شده و 5 نمک تصفیه نشده آزمایش شد. دقت آزمایشات با تکرار 5 نمونه صورت گرفت. اندازه گیری فلزات سنگین با دستگاه جذب اتمی کوره گرافیتی و اسپکترومتر نشر اتمی با پلاسمای کوپل القایی صورت گرفت و سپس با آزمون آماری t-test توصیف گردیدند. یافته‌ها: میانگین غلظت کادمیوم، سرب، آرسنیک، جیوه، مس و روی در نمک‌های تصفیه شده به ترتیب15/0، 57/0، 69/0، 061/0، 87/0 و µg/g 34/6 و در نمک‌های تصفیه نشده برابر16/0، 61/0، 63/0، 058/0، 86/0 و µg/g 53/7 بود. نتیجه گیری: میانگین غلظت آرسنیک و جیوه در نمونه‌های نمک موجود، بالا بوده و از مقادیر استاندارد بیشتر می باشد، اما میانگین غلظت سایر فلزات و استاندارد آنها در نمک‌های تصفیه شده و تصفیه نشده از این حدود پایین تر است. همچنین بین میانگین غلظت نمکهای تصفیه شده و نشده تفاوت معنی داری وجود ندارد. مقدار دریافت هفتگی قابل تحمل (PTWI) فلزات سنگین از طریق نمک طعام پایین تر از حدود استاندارد برآورد گردید. با این وجودکاربرد روش‌های مناسب حذف آلاینده از نمک ضرورت دارد.

Title: بررسی غلظت ذرات معلق در هوای داخل کلاس و هوای بیرون و عوامل تأثیرگذار آن در مدارس راهنمایی و دبیرستان شهر یزد

http://tbj.ssu.ac.ir/article-1-1773-fa.html

چکیده مقدمه: آلودگی محیط‌های عمومی مانند مدارس به ذرات معلق در هوا اثرات نامطلوبی بر سلامتی معلمین و دانش آموزان که بخش قابل توجه ای از وقت خود را در مدرسه سپری می‌کنند می‌گذارد. هدف از این مطالعه اندازه گیری غلظت ذرات معلق در هوای کلاس و هوای بیرون کلاس در مدارس شهر یزد و تعیین پارامتر‌های تأثیرگذار بر آنها می‌باشد. روش بررسی: مطالعه بصورت تحلیلی- مقطعی در 20 مدرسه دخترانه و پسرانه مقاطع راهنمایی و دبیرستان شهر یزد در زمستان 91 انجام شد. از دستگاه مونیتورینگ گرد و غبار محیطی مدل 5000HAZ-DUST EPAM برای اندازه گیری ذرات 10PM، 2.5PM و 1PM استفاده شد. سپس داده‌ها با آزمون‌ها همبستگی، مقایسه میانگین و رگرسیون مورد بررسی قرار گرفتند. نتایج بدست آمده با استاندارد سازمان جهانی بهداشت و EPA مقایسه شد. یافته‌ها: متوسط غلظت ذرات10PM، 2.5PM و 1PM در هوای داخل کلاس نسبت به هوای آزاد، بالاتر بود. وضعیت کیفی هوای داخل و هوای آزاد مدارس از لحاظ شاخص کیفیت هوا(AQI Calculator) برای 10PM متوسط، برای 2.5PM ناسالم را برای گروه‌های حساس نشان داد. رابطه معنی‌داری بین غلظت ذرات در هوای داخل و خارج دیده شد(05/0>P). میانگین نسبت ذرات هوای داخل به هوای خارج مدارس(I/O) به ترتیب 68/1، 31/1، 46/1 بود. نتیجه‌گیری: ارتباط معنی‌داری که بین غلظت ذرات معلق در هوای خارج و هوای داخل مدارس وجود داشت، نشان دهنده نفوذ ذرات به داخل کلاس بود. از این رو، برای مدارس مختلف بهره‌گیری از سیستم‌های مناسب تهویه در کاهش میزان آلودگی هوای داخل کلاس، موثر می‌باشد. این مقاله برگرفته از پایان نامه دانشجویی می‌باشد.

Title: ارزیابی عملکرد مدیریت ایمنی و بهداشت حرفه‌ای با استفاده از مدل مدیریت ایمنی کیفیت جامع (TQSM)

http://tbj.ssu.ac.ir/article-1-1772-fa.html

مقدمه: یکی از مدل‌های موجود جهت ارزیابی عملکرد مدیریت ایمنی و بهداشت حرفه‌ای، مدل مدیریت ایمنی کیفیت جامع (TQSM) می‌باشد. هدف از این مطالعه،ارزیابی عملکرد مدیریت ایمنی و بهداشت حرفه‌ای با استفاده از مدل TQSM در یک شرکت تولیدی می‌باشد. روش بررسی: این تحقیق از نوع مطالعات توصیفی- تحلیلی می‌باشد. جامعه آماری شامل16 نفر از مدیران، سرپرستان و اعضاء کمیته حفاظت فنی و بهداشت کار هستند. که به سؤال‌های پرسشنامه TQSM که شامل چهار برنامه TQM، VPP، PSM و ISO9001 می‌باشد در قبل و بعد از استقرار سری ارزیابی ایمنی و بهداشت شغلی 18001(OHSAS18001) پاسخ دادند. در نهایت سطح هر کدام از برنامه‌ها مشخص و وضیعت TQSM در قبل و بعد از استقرار OHSAS18001 تعیین شد. یافته‌ها: نتایج نشان داد امتیاز کسب شده توسط شرکت قبل از استقرار OHSAS18001، 7/43 از 312 امتیاز بوده است. بعد از استقرار OHSAS18001در شرکت و دریافت گواهینامه مربوطه، مجموع امتیاز برنامه ایمنی که شرکت توانست کسب کند 12/127 امتیاز از 312 امتیاز بوده است که نسبت به قبل 42/83 امتیاز (8/26 درصد امتیاز کل) ارتقا داشت. آزمون مقایسه میانگین‌ها نشان داد اختلاف میانگین امتیازات TQSM قبل و بعد از استقرار OHSAS18001 معنی‌دار است (05/0>p). نتیجه‌گیری: این مطالعه نشان داد مدل مدیریت ایمنی کیفیت جامع بدلیل توانایی آن در ارزیابی کمی عملکرد سیستم، مدل مناسبی جهت پایش عملکرد سیستم مدیریت ایمنی و بهداشت حرفه‌ای می‌باشد.

Title: بررسی میزان سرب و کادمیوم در نان‌های تولیدی در شهرستان یزد

http://tbj.ssu.ac.ir/article-1-1776-fa.html

برای تجزیه و تحلیل داده‌ها از بسته نرم افزاری SPSS نسخه 17 استفاده شد. سطح معنی‌داری P کمتر از05/0 در نظر گرفته شد. یافته‌ها: میانگین مقدار سرب در نمونه‌‌ها 05/99 میکروگرم بر کیلوگرم و کادمیم49/7 میکروگرم بر کیلوگرم بدست آمد. میزان سرب در نان سنگک بیشتر و در نان فانتزی کمتر از بقیه انواع نان بود و میزان کادمیم در نانها تفاوت چشمگیری نداشت. میزان سرب در نانهای با حرارت مستقیم بیشتر و بین میزان کادمیم در نانهای با حرارت مستقیم و غیر مستقیم اختلاف زیادی وجود نداشت و در تمامی نمونه‌ها مقادیر سرب و کادمیم از حد مجاز تعیین شده، کم‌تر بود. نتیجه گیری: بر اساس نتایج این پژوهش وبا در نظر گرفتن اینکه مصرف سرانه نان در ایران حدود160 کیلوگرم است، انتظار می‌رود که میزان دریافت هفتگی سرب و کادمیم از طریق نان برای مصرف کنندگان شهرستان یزد در حد قابل قبولی باشد. اما باید این مقدار کاهش یابد تا از بروز خطرات احتمالی فلزات سنگین سرب و کادمیم جلوگیری شود. این مقاله حاصل پایان نامه کارشناسی ارشد دانشکده بهداشت دانشگاه علوم پزشکی شهید صدوقی یزد است.

Title: مقایسه سلامت روان دانشجویان دختر ورزشکار و غیر ورزشکار دانشگاه علوم پزشکی شهید صدوقی یزد

http://tbj.ssu.ac.ir/article-1-1775-fa.html

چکیده مقدمه: در بیست سال گذشته توجه بسیاری به مسائل روان‌شناسی ، روان‌درمانی با فعالیت بدنی شده است. با توجه به اهمیت سلامت روان دانشجویان، این مقاله به مقایسه سلامت روان دانشجویان دختر ورزشکار و غیر ورزشکار دانشگاه علوم پزشکی شهید صدوقی یزد پرداخته است. روش بررسی: در این مطالعه توصیفی و تحلیلی تعداد 200 نفر از دانشجویان دختر دانشگاه علوم پزشکی شهید صدوقی یزد انتخاب شدند. گروه مورد را دانشجویان دختر ورزشکار و گروه شاهد را دانشجویان دختر غیرورزشکار تشکیل دادند. ابزار سنجش پرسشنامه GHQ28 سوالی به همراه سوالاتی از ویژگی‌های دموگرافیک بود. داده‌‌ها به وسیله نرم افزار spss نسخه6 و با استفاده از فراوانی و انحراف معیار و آزمون آماری تی تست و کای اسکوار مورد تجزیه و تحلیل قرارگرفت. یافته‌ها: داده‌ها نشان داد که دانشجویان دختر ورزشکار در میانگین نمره سلامت عمومی ‌تفاوت معنی‌داری با دانشجویان غیر ورزشکار داشتند(05/0>p). بیشترین میانگین نمره مربوط به زیرمقیاس افسردگی در بین دانشجویان ورزشکار و اختلال در کارکرد اجتماعی دختران غیرورزشکار و کمترین میانگین نمره مربو ط به زیر مقیاس اضطراب در هردوگروه دانشجویان بود. همچنین میانگین نمره سلامت عمومی‌دانشجویان غیرورزشکار از نقطه برش23 بالاتر بود. نتیجه‌گیری: با توجه به نتایج این مطالعه می‌توان گفت ،سلامت عمومی‌دانشجویان ورزشکار از دانشجویان غیرورزشکار بهتر بود، اما مساله قابل توجه، بالابودن میزان افسردگی در هردو گروه دانشجویان دختر و در خطر بودن سلامت عمومی ‌دانشجویان دختر غیر‌ورزشکار در این مطالعه است.

Title: مطالعه اثر تابش پرتو الکترونی و تعیین دوز موثردر نابودی انگل لینگواتولا سراتا جدا شده از فراورده‌های دامی

http://tbj.ssu.ac.ir/article-1-1778-fa.html

چکیده مقدمه: بیماری‌های انگلی منتقله از طریق غذا، در اکثر نقاط جهان شایع بوده و مشکلات بهداشتی مهمی ‌را سبب می‌شوند. لینگواتولا سراتا انگلی زئونوز و عامل ایجاد لینگواتولیازیس انسانی به واسطه مصرف احشاء دامی‌ خام و نیم پخته  آلوده به نوچه این انگل می‌باشد. هدف اصلی این تحقیق، تعیین اثر تابش پرتو الکترونی در نابودی انگل لینگواتولا سراتا جدا شده از فرآورده‌های دامی‌ بود. روش بررسی: نوچه‌های انگل لینگواتولا سراتا، تحت اشعه دهی با پرتوالکترونی در دوزهای 1، 2، 3 و 5 کیلوگری(15 نوچه در سه تکرار 5تایی برای هر دوز)  قرار گرفتند. با بررسی حرکت نوچه‌ها در زیر استریومیکروسکوپ زمان مرگ نوچه‌ها مورد بررسی قرار گرفته و با گروه شاهد نگهداری شده در دمای 4 درجه سانتی گراد مقایسه گردید. آنالیز داده‌ها با آزمون‌های t- test و ANOVA در سطح معنی‌داری 05/0 انجام شد. نتایج: مقایسه دو گروه شاهد و تیمار نشان داد که از نظر آماری، اختلاف معنی‌داری در زمان مرگ نوچه‌ها وجود دارد(05/0>P). همچنین اختلاف آماری ‌معنی‌داری بین دوز‌های 1، 2 و 3 کیلوگری با دوز 5 کیلوگری از نظر سرعت کشندگی وجود داشت(05/0>P). نتایج نشان داد که حداقل دوز نابودی انگل لینگواتولا سراتا 1 کیلوگری  بوده و دوز5 کیلوگری، سبب مرگ سریعتر نوچه‌ها گردید. نتیجه گیری: با توجه به حساسیت نوچه انگل لینگواتولا سراتا  به پرتو الکترونی، در آینده می‌توان از پرتوالکترونی جهت ارتقاء ایمنی فراورده‌های دامی‌استفاده نمود. این مقاله حاصل پایان نامه دانشجوی کارشناسی ارشد گروه بهداشت و ایمنی مواد غذایی دانشکده بهداشت دانشگاه علوم پزشکی و خدمات بهداشتی درمانی شهید صدوقی یزدمی‌باشد.

Title: بررسی میزان آگاهی، نگرش و عملکرد متصدیان کشت گلخانه‌ای شهرستان یزد در رابطه با مخاطرات بهداشتی کاربرد سموم در سال 1391

http://tbj.ssu.ac.ir/article-1-1777-fa.html

مقدمه: آفت کش‌های مورد استفاده در کشاورزی، یکی از منابع مهم آلودگی محیط زیست هستند که بر سلامت موجودات زنده از جمله انسان­ها، تأثیر منفی دارند. هدف از انجام این مطالعه، بررسی میزان آگاهی، نگرش و عملکرد متصدیان کشت گلخانه­ای شهرستان یزد در رابطه با مخاطرات بهداشتی کاربرد سموم در سال1391 بوده است. روش بررسی: این مطالعه، یک مطالعه توصیفی(مقطعی) است که جمعیت مورد مطالعه در آن تحقیق، متصدیان گلخانه‌های فعال شهرستان یزد بودند. در این مطالعه به صورت تصادفی، تعداد250 نمونه از2233  متصدی گلخانه انتخاب گردید و سپس با استفاده از پرسشنامه آگاهی، نگرش و رفتارهای کارگران، مورد سنجش قرار گرفت. پس از جمع آوری داده­ها و کدگذاری­، داده‌ها وارد نرم افزار SPSS شده و جهت مقایسه میانگین داده از آزمونT-test  و ANOVA استفاده شد. یافته‌ها: نتایج مطالعه حاضر نشان داد که از کل متصدیان گلخانه، تعداد134 نفر(6/53 درصد) آموزش‌های مرتبط با کاربرد سموم را دیده‌بودند. همچنین نتایج نشان دادکه با توجه به نمره آگاهی که حداکثر13 می‌باشد، هیچ کدام از متصدیان، نمره کامل را دریافت نکردند. همچنین میزان نگرش متصدیان گلخانه نسبت به کاربرد سموم، در حد بالا و به تعداد 243 نفر می‌باشد. میزان عملکرد 138 نفر از متصدیان گلخانه، در حد متوسط، قرار داشت. نتیجه گیری: با وجود این که اطلاعات متصدیان گلخانه مورد مطالعه در بعضی زمینه‌ها، نظیر اقدامات احتیاطی هنگام کار با سموم به نسبت خوب می‌باشد، در بعضی زمینه‌ها مانند استفاده از ماسک تنفسی مناسب برای سم پاشی و لباس کار مخصوص کافی نبوده که نشانگر ضرورت تدوین و اجرای برنامه آموزشی، تخصصی است. این مقاله برگرفته از پایان نامه دانشجویی دانشگاه علوم پزشکی و خدمات بهداشتی درمانی شهید صدوقی یزد می‌باشد.

Title: بررسی کیفیت زندگی در بیماران مبتلا به دیابت نوع دو و مقایسه آن با افراد سالم و ارائه یک مدل ساختاری

http://tbj.ssu.ac.ir/article-1-1779-fa.html

مقدمه: یکی از مفاهیم بسیارمهم دردیابت ملیتوس به عنوان یک بیماری مزمن، کیفیت زندگی است که بر عملکرد فردی واجتماعی اثرات گسترده ای دارد.  با توجه به عوارض کوتاه مدت، بلندمدت وهزینه‌های بسیارزیاداین بیماری، مطالعه حاضر با هدف تعیین کیفیت زندگی این بیماران در مقایسه با افراد غیر دیابتی انجام شدودر نهایت یک مدل ساختاری ارایه گردید.  روش بررسی: این مطالعه به صورت تحلیلی بر روی 250بیماردیابتی و250 بیمار غیر دیابتی در شهر یزد انجام شد. در این مطالعه از ابزارwhoqol-bref  استفاده شد این ابزار شامل 26 سوال و 4 حیطه سلامت جسمانی، سلامت روانی، سلامت اجتماعی و سلامت محیط  می‌باشد. داده‌ها با استفاده از نرم افزار (16) spss و (21) amos  تحلیل گردید. یافته‌ها: بین کیفیت زندگی دو گروه بیماران دیابتی و افراد سالم با001/0p< تفاوت معنی‌داری وجود داشت و میانگین تمام حیطه‌های مختلف پرسشنامه در بیماران دیابتی پایین تر از افراد سالم بود. ضرایب مسیر در معادله ساختاری ارایه شده، همگی با 001/0 P <معنی‌دارشدند. ضریب مسیر بین سلامت اجتماعی و سلامت محیط با سلامت روان به ترتیب برابر با 62/0 و76/ بود. ضریب مسیر بین سلامت روان وسلامت جسمانی برابر با 96 /0 شد. 86 درصدازتغییرات متغیرسلامت جسمانی توسط متغیر سلامت روان و 96 درصداز تغییرات متغیرسلامت روان توسط سلامت اجتماغی وسلامت محیط تبیین شده است. نتیجه گیری:دیابت بیماری مزمنی است که کیفیت زندگی بیماران را به شدت تحت تأثیر قرار می‌گیرد.هرگونه تلاش در مدل‌های ساختاری پیشبینی کننده سلامت جسمانی و کیفیت زندگی، نقشی معنی دار در حل دشواریهای این بیماران ایفا می‌کند.

Title: مقایسه حمایت اجتماعی ادراک شده و امید به زندگی در سالمندانِ مقیم و غیر مقیمِ خانه‌های سالمندانِ استان یزد

http://tbj.ssu.ac.ir/article-1-1780-fa.html

چکیده مقدمه: سالمندی، دوران حساسی از زندگی بشر است و توجه به مسائل و نیازهای این مرحله، ضرورتی اجتماعی است. پژوهش‌ها نشان داده اند که زندگی با سایر اعضای خانواده بر سلامت جسمانی و روانی سالمندان تأثیر مثبت دارد. این بررسی با هدف مقایسه حمایت اجتماعی ادراک شده و امید به زندگی در سالمندانِ مقیم و غیر مقیمِ خانه‌های سالمندانِ استان یزد انجام گردید. روش بررسی: این پژوهش از نوع توصیفی- پیمایشی می‌باشد. جامعه پژوهش حاضر، شامل تمامی سالمندانِ مقیم و غیر مقیمِ خانه‌های سالمندانِ استان یزد بود که از این جامعه، نمونه ای به حجم 117 نفر با روش  نمونه‌گیری در دسترس انتخاب شدند. برای سنجش متغیرهای پژوهش از پرسشنامه حمایت اجتماعی و پرسشنامه امید به زندگی استفاده شد. داده‌ها با استفاده از آمار توصیفی، ضریب همبستگی پیرسون و تحلیل واریانس عاملی مورد تجزیه و تحلیل قرار گرفتند. یافته‌ها: یافته‌های پژوهش نشان داد بین دو متغیر حمایت اجتماعی ادراک شده و امید به زندگی در سالمندان مقیم و غیر مقیم رابطه مستقیم و معنی‌دار وجود دارد (01/0>p). همچنین، بین نمره حمایت اجتماعی و امید زندگی دو گروه از سالمندان مقیم و غیر مقیم، سالمندان زن و مرد و افراد در خرده گروه‌های وضعیت تأهل، تفاوت معنی‌دار وجود دارد (01/0>p). بحث و نتیجه گیری: بر اساس یافته‌های این پژوهش، به خانواده‌ها و فرزندان توصیه شده است حتی‌الامکان جهت ارتقاء سلامت اجتماعی و روانی سالمندان، ایشان را در منازل خود تحت مراقبت و خدمت رسانی قرار دهند.

Title: نقش خلاقیت سازمانی بر کارآفرینی سازمانی (مطالعه موردی: کارکنان اداری دانشگاه علوم پزشکی شهید بهشتی تهران)

http://tbj.ssu.ac.ir/article-1-1781-fa.html

مقدمه: سازمانهای عمومی‌ برای ارتقای میزان خدمات رسانی به مشتریان و حفظ جایگاه خود باید به تدوین و اجرای  برنامه های راهبردی بپردازند که از جمله آن، راهبرد کارآفرینی است. این راهبرد مستلزم عوامل و شرایط متعددی است که هدف مطالعه حاضر سنجش نقش خلاقیت سازمانی بر کارآفرینی سازمانی در دانشگاه علوم پزشکی شهید بهشتی تهران پرداخته شده است. روش بررسی: مطالعه حاضر از نوع توصیفی- همبستگی بوده که به شیوه نمونه گیری تصادفی - طبقه ای تعداد 347 نفر از کارکنان اداری دانشگاه علوم پزشکی شهید بهشتی تهران از طریق دو پرسشنامه خلاقیت سازمانی (1998 Dorabjee et.al,) و کارآفرینی سازمانی ( 2007Leonidas & Vassillis, ) مورد مطالعه قرار گرفتند. برای تجزیه و تحلیل داده ها از آزمون ضریب همبستگی پیرسون و رگرسیون چندگانه با استفاده از نرم افزار  SPSSنسخه 17 استفاده شد. یافته‌ها: بین خلاقیت سازمانی (و مؤلفه های آن) و کارآفرینی سازمانی رابطه مثبت و معنی‌داری وجود داشت (01/0 p<). نتایج رگرسیون چندگانه نشان داد که از بین مؤلفه های خلاقیت سازمانی، مؤلفه‌های چالش، آزادی، اعتماد، خوش خلقی، تضادها و خطرپذیری توان پیش بینی کارآفرینی سازمانی را دارند. نتیجه‌گیری: در دنیای پرتلاطم و رقابتی امروزی سازمانها از جمله دانشگاه‌های علوم پزشکی نیازمند کارآفرینی و کارکنان کارآفرین می‌باشند که یکی از الزامات تحقق این هدف، گسترش رویکردهای خلاقانه و نوآورانه سازمانی می‌باشد زیرا این مطالعه نشان داد که با افزایش خلاقیت سازمانی کارکنان، کارآفرینی سازمانی آنان نیز افزایش می‌یابد.

Title: بررسی کارایی نانولوله‌های کربنی چند دیواره در حذف رنگ اسید بلاک 1 از محلول‌های آبی

http://tbj.ssu.ac.ir/article-1-1783-fa.html

چکیده مقدمه: پساب رنگی برخی صنایع شامل تعدادی ترکیبات سرطان زا، سمی ‌و یا جهش زا می‌باشد. بنابراین آلاینده‌های رنگی فاضلاب این نوع صنایع باید قبل از تخلیه به محیط، با روش مناسب تصفیه گردند. هدف از انجام این پژوهش بررسی کارآیی نانو تیوب کربن چند دیواره در حذف رنگ اسید بلاک 1 از فاضلاب رنگی است. روش بررسی: این پژوهش یک مطالعه آزمایشگاهی می‌باشد که در سیستم ناپیوسته انجام شده و از نانو تیوب کربن چند دیواره به عنوان جاذب برای حذف رنگ استفاده شده است. دراین مطالعه عوامل مختلف تأثیرگذار روی میزان حذف رنگ مانند دوز جاذب، غلظت اولیه رنگ، زمان تماس و pH مورد بررسی قرار گرفت. یافته‌ها: نتایج نشان داد که 3=pH مناسبترین pH، برای حذف رنگ است و زمان تعادل برای جذب رنگ اسید بلاک 1روی جاذب برابر60 دقیقه بود. با افزایش غلظت رنگ، سرعت حذف رنگ کاهش یافت. افزایش مقدار جاذب باعث افزایش راندمان حذف رنگ شد در دوز 600 میلی گرم بر لیتر راندمان حذف برای رنگ با غلظت30 و 50 میلی‌گرم بر لیتر به ترتیب برابر86/98% و62/94% درصد به دست آمد. همچنین حذف رنگ اسیدبلاک1 از مدل ایزوترمی‌لانگمویرو سینتیک درجه دومتبعیت کرد. نتیجه گیری: نتایج مطالعات جذب نشان داد که با افزایش زمان تماس و دوز جاذب و کاهش pH، راندمان حذف رنگ افزایش می‌یابد. بر اساس نتایج این تحقیق، نانو لوله‌های کربنی چند جداره به عنوان یک جاذب موثر برای حذف رنگ‌های آزو از فاضلاب می‌توانند استفاده شوند.

Title: بررسی کارایی نانولوله‌های کربنی چند جداره اصلاح شده در حذف وانادیوم از پساب نفتی

http://tbj.ssu.ac.ir/article-1-1782-fa.html

مقدمه: وانادیوم و ترکیباتش از مهم­ترین آلاینده­های محیط زیست بوده که به دلیل اثرات مخرب بر سلامتی انسان‌ها و بهداشت محیط، توسعه روش­های جدید برای حذف آن­ از اهمیت فوق­العاده برخوردار است. لذا این مطالعه با هدف بررسی کارایی حذف وانادیوم از پساب جمع آوری شده در مخازن جداکننده پالایشگاه نفت امام خمینی(ره) شازند اراک، توسط جاذب نانو لوله کربنی چند جداره اصلاح شده انجام شد. روش بررسی: این پژوهش با استفاده از روش Batch در محیط آزمایشگاه بر روی پساب نفتی به ترتیب با غلظت­های 3، 5، 7، 9، 11، 13، 15، 17، 19، 21، 23 میلی­گرم در لیتر وانادیوم انجام و تأثیر متغیرهای مقدار جاذب، غلظت اولیه محلول وانادیوم، pH، دما و زمان تماس بر کارایی حذف وانادیوم توسط جاذب، ارزیابی شد. به منظور بهبود عملکرد جاذب، از فرآیند اکسیداسیون توسط اسید نیتریک استفاده و طیف IR و تصویر  SEMجاذب، تهیه شد. همچنین داده‌ها با ایزوترم­های لانگمویر، فروندلیخ و تمکین مطابقت داده شدند. یافته‌ها: نتایج، نشان داد که کارایی حذف وانادیوم از پساب نفتی توسط جاذب در دمای 20 درجه سانتی­گراد، زمان 130 دقیقه، مقدار جاذب برابر با 03/0 گرم و pH بهینه برابر با 5، به 99 درصد رسیده و جذب تعادلی برای جاذب، منطبق بر ایزوترم فروندلیچ (995/0 R2=) بوده است. نتیجه گیری: نتایج پژوهش نشان داد که نانو لوله کربنی چند جداره اصلاح شده، می‌تواند به عنوان یک جاذب موثر برای حذف فلزات سنگین سمی‌از پساب صنعتی، مورد استفاده قرار گیرد.

Title: شیوع و عوامل مرتبط با دیابت نوع دو در شهرستان میبد -1390

http://tbj.ssu.ac.ir/article-1-1785-fa.html

چکیده مقدمه: دیابت نوع دو شایعترین فرم بیماری دیابت می باشد. عواملی همچون سابقه ژنتیکی، اضافه وزن و چاقی و فشار خون بالا از عوامل خطر مهم این بیماری می باشند. این مطالعه با هدف تعیین شیوع و عوامل مرتبط با آن در افراد بالای 30 سال شهرستان میبد انجام گرفته است. روش بررسی: این مطالعه از نوع توصیفی- مقطعی می باشد. در این مطالعه از داده‌های بدست آمده از طرح اجرایی غربالگری دیابت و فشارخون به تعداد 14276 نفر که در بین افراد 30 ساله و بالاتر شهرستان میبد به اجرا در آمده، استفاده شده است. تجزیه و تحلیل‌های مورد نیاز با استفاده از آزمون آماری کای دو و در محیط نرم افزار آماری SPSS انجام شده است. یافته‌ها: در این مطالعه شیوع افراد مبتلا به دیابت نوع دو و افراد قرار گرفته در مرحله پره دیابتیک به ترتیب 12 و 5/9 درصد بدست آمد و به طور معنی‌داری شیوع بیماران در شهر بیشتر از روستا، در زنان بیشتر از مردان و در افراد مسن تر بیشتر از سنین پایین تر بود. همچنین این شیوع درافراد قرار گرفته در گروه‌های دارای اضافه وزن، چاق، فشارخون بالا، سابقه دیابت در اطرافیان، سابقه دیابت بارداری، سابقه سقط، مرده زایی و یا نوزاد بالای 4000 گرم نیز بیشتر از دیگران بود. نتیجه گیری: با توجه به شیوع بالای دیابت نوع دو در این شهرستان، لازم است برنامه‌های پیشگیری و تشخیص زودرس این بیماری تقویت شوند و در این خصوص باید بر زنان، افراد با سنین بالاتر و ساکنین شهر تاکید بیشتری شود و افراد دارای سابقه دیابت در اطرافیان، سابقه دیابت بارداری، سابقه سقط، مرده زایی یا تولد نوزاد با وزن بالای 4000 گرم در اولویت قرار گرفته و با دقت بیشتر بررسی شوند.

Title: استفاده تلفیقی از مدلهای AHP و TOPSIS در مکان‌یابی محل دفن بهداشتی پسماندهای شهری (مطالعه موردی: شهرلالی)

http://tbj.ssu.ac.ir/article-1-1784-fa.html

مقدمه: انتخاب محل دفن مناسب برای دفع پسماندهای جامد شهری، یکی از پیچیده‌ترین و دشوارترین موارد در مدیریت پسماندها بشمار میرود زیرا باید عوامل و معیارهای متعدد زیست محیطی، فنی و اقتصادی و اجتماعی و فرهنگی، همزمان مورد توجه و بررسی قرار گیرد. هدف از انجام این مطالعه، انتخاب محل دفن مناسب برای پسماندهای شهری شهر لالی می‌باشد. روش بررسی: در این تحقیق، برای انتخاب محل دفن بهداشتی از سامانه اطلاعات جغرافیایی و از مدلهای AHP وTOPSIS  استفاده شد. با در نظر گرفتن 11 معیار و با استفاده از مدلAHP ، 6 ناحیه جهت دفن بهداشتی پسماندها شناسایی شده و سپس این مناطق با استفاده از روش  TOPSIS و با استفاده از 8 معیار جدید که بر اساس مطالعه پروژه‌های مشابه و بازدید میدانی و صحبت با کارشناسان مربوطه تعیین شدند به ترتیب اولویت رتبه بندی شده و در نهایت محل مناسب برای دفن بهداشتی شناسایی گردید. یافته‌ها: با عنایت به اینکه در این پژوهش از تکنیک AHP، جهت وزن دهی و از مدل TOPSIS که یکی از روش‌های تصمیم گیری چندمعیاره می‌باشد، به منظور رتبه بندی گزینه‌ها استفاده گردید، در نهایت گزینه شماره 4 (واقع در اراضی ملی روستای خواجه آباد) در رتبه اول قرار گرفت و به عنوان محل دفن پیشنهادی پسماندهای شهر لالی معرفی شد. نتیجه‌گیری: استفاده ترکیبی از مدلهای AHP و TOPSIS می‌تواند به عنوان یک روش مناسب برای شناسایی محلهای دفن بهداشتی در کشورمان مورد توجه قرار بگیرد.
